# Exploring the relationships between socio-economic indicators and student enrollment in higher education institutions of Pakistan

**DOI:** 10.1371/journal.pone.0261577

**Published:** 2021-12-22

**Authors:** Syeda Mubashira Batool, Zhimin Liu

**Affiliations:** 1 College of Public Administration, Nanjing Agricultural University, Nanjing, Peoples Republic of China; 2 Higher Educational Institute, Nanjing Agricultural University, Nanjing, Peoples Republic of China; Ghazi University, PAKISTAN

## Abstract

Higher education is considered as the engine of the economic development of a country due to its role in cultivating human capital. The provision of higher education is regarded as a productive investment in human capital for improving nation’s productive capacity. However, there is a large gap in enrollment between students of different socioeconomic statuses. The ever-widening socio-economic inequalities between individuals from disadvantaged and advantaged backgrounds make the government’s efforts to enroll a higher number of students to pursue higher education challenging in developing countries, as the students’ socioeconomic status plays a decisive role in their priority to attain higher education. This study anticipated quantifying the impact of socio-economic indicators and underlying situations on students’ enrollment in higher education in Pakistan. A descriptive study, involving correlation, principal component analysis (PCA), clustering, and stepwise regression using 15-years data of enrollment and socio-economic indicators was conducted. The correlation between different socio-economic indicators and students’ enrollment was positive and highly significant (0.73 to 0.99), except for the unemployment rate (- 0.39 to -0.57). PCA showed that the first two components were the most influential with 93.85% of the total variation. Enrollment (total and male) showed significant relationships with general government expenditure and unemployment rate, while female enrollment showed a significant relationship with general government expenditure. Findings revealed that socio-economic factors can serve as a significant predictor of students’ enrollment in higher education. The minimum values of correlation coefficient (R) and adjusted R^2^ for enrollment were ranged from 0.875 to 0.748 (female enrollment), while maximum values (0.987 to 0.993 and 0.973 to 0.983), respectively were observed for total enrollment. The findings will assist educationists, social scientists, and policymakers to better understand the association between socio-economic indicators and student enrollment in higher education for formulating future education policies for enhancing enrollment in higher education.

## 1. Introduction

Higher education plays an imperative role in the economic development of a country through talent cultivation. The provision of higher education is considered a productive investment in human capital, which refers to the knowledge, skill sets, and experience individuals have in an economy. Therefore, governments aim to ensure a high participation of students in higher education, and participation in higher education is gradually increasing over time worldwide due to its significance for the social and economic progression of a country [1]. However, there is a large gap in enrollment between students from disadvantaged and advantaged backgrounds, as students from disadvantaged socio-economic backgrounds are less likely to attend higher education institutions as compared to those from advantaged socio-economic backgrounds despite having similar levels of prior academic achievement and skills [2]. Low human capital, lower preferences for education, and high sensitivity to costs are among the key factors for the low participation in higher education by students from disadvantaged socio-economic backgrounds [3]. Higher education enhances human capital and labor input quality by facilitating economic growth owing to higher wages associated with higher education [4, 5]. Although, students from low-income families can improve their socio-economic status, yet they invest less in education due to the socio-economic condition of their families. Therefore, governments generally implement financial aid programs to increase the participation of these students.

Human capital impacts economic growth and can help to grow the economy by expanding the knowledge and skills of individuals. There is a strong correlation between human capital and economic growth. However, the reports regarding the association between the expansion of higher education and the economic status of a country are contradicting. The expansion in higher education coincided with the economic decline and a rise in graduate unemployment [6], while another study argued that the government used higher education as a labor market tool against unemployment [7, 8]. The reason behind this contradiction is that if the unemployment rates or the tension in the labor market are too high in an economy, the higher education system has the potential to easily decrease the unemployment rate by pulling the idle workforce back into the higher education system.

The importance of higher education for economics mainly stems from its ability to create and/or accumulate human capital and increase the aggregate productivity level of the economy [9, 10]. Thus, the economy can produce more efficiently with an increase in productivity level [6, 11]. These effects of human capital have led countries to invest in higher education resulting in an increase of higher education institutions and the student number worldwide. An association between the participation of students in higher education and the socio-economic status of their family and country has been reported [2, 3]. Additionally, overall increased participation in higher education over time has also been documented, however, this increase was smaller for low-income families [12]. The quality of mass higher education varies in the extent of upward social mobility from low socio-economic status backgrounds. Family income highly impacts enrollment at all levels. Privileged groups benefit from valuable resources and access to quality education [13].

Countries with high public spending and low public spending have a strong association with the percentages of enrolled students [14, 15]. The gross tertiary enrollment ratio estimated by UNESCO in developed countries is (75.03) while in developing countries is (31.22). Additionally, it is 7.46, 34.54, and 75.14598 in low-income, middle-income, and high-income countries, respectively [16]. Attempts have been made to examine the impact of socio-economic factors on enrollment in higher education [17], the increasing role of private associations, the effect of public funding on enrollment in higher education, faculty ratio, literacy rate, and gender parity in enrollments [18–21]. Studies also reported increasing trends of female students enrolling and obtaining degrees as compared to male students in most of the countries [22]. Studies on the association between socioeconomic factors and enrollment indicated that enrollment in higher education is generally dependent on the socio-economic conditions of students and a country [23]. Gross enrollment in higher education in the USA, Finland, and South Korea was above 70%, while in Kenya and Ethiopia it was 1% [17]. Family income and parental education also affect educational attainment [23]. Income effect on higher education enrollment is positive, and students from poorer backgrounds may not be able to invest in their education [2, 3]. So, governments are required to carry on bearing great accountability for financing higher education to overcome socio-economic inequalities between lower and upper-income scholars in pursuing higher education [24, 25]. The financial support, per capita income, and future earnings also have a positive weight on male participation in education [26]. There are now a record number of people enrolled, studying at a diverse set of higher education institutions. Improvements have been made to ensure that students from disadvantaged schools or backgrounds are given a fair chance to study for a degree.

A strong relationship between economic growth and higher education suggests that these variables are necessary for each other. A highly subsidized education system needs to be introduced to increase enrollment in higher education [27]. The higher government spending and the number of schools demonstrated a positive impact on student enrollment in Pakistan [28]. However, poverty and household income are reported to influence primary school enrollment [29]. Lower student enrollment in developing countries cannot be attributed due to a smaller number of institutes as other factors such as government disbursement, employment rate, and expenses on health sector is reported to increase enrollment in all levels of education in Pakistan [30, 31]. Despite, several studies explaining the relations between demographic, cultural, political, and educational factors including institutions and teachers in the education system of Pakistan. Yet, no attempt has been made to examine the impact of socio-economic factors (general government expenditure, gross domestic production (GDP) per capita, GDP at market price, unemployment rate, and per capita income) on enrollment (total, male and female) in higher education institutions of Pakistan.

The concept of human capital has been brought to the forefront of many discourses in the field of the economic development of a society. This study hypothesized that higher education attainment and socio-economic status of students’ families and country have a close association. This study emphasized the human capital theory while focusing on the sociology of education. The present study was endeavored to investigate the theoretical and empirical connections between student enrollment in higher education and socio-economic status of individuals by exploring the impact of socio-economic factors (general government expenditure, GDP per capita, GDP at market price, unemployment rate, and per capita income) on enrollment (total, male, female) in higher education institutions in Pakistan. The findings will assist educationists, social scientists, and policymakers to better understand the association between socio-economic indicators and student enrollment in higher education for formulating future education policies for increasing enrollment in higher education in Pakistan.

## 2. Methodology

### 2.1 Research design

**Fig 1:**
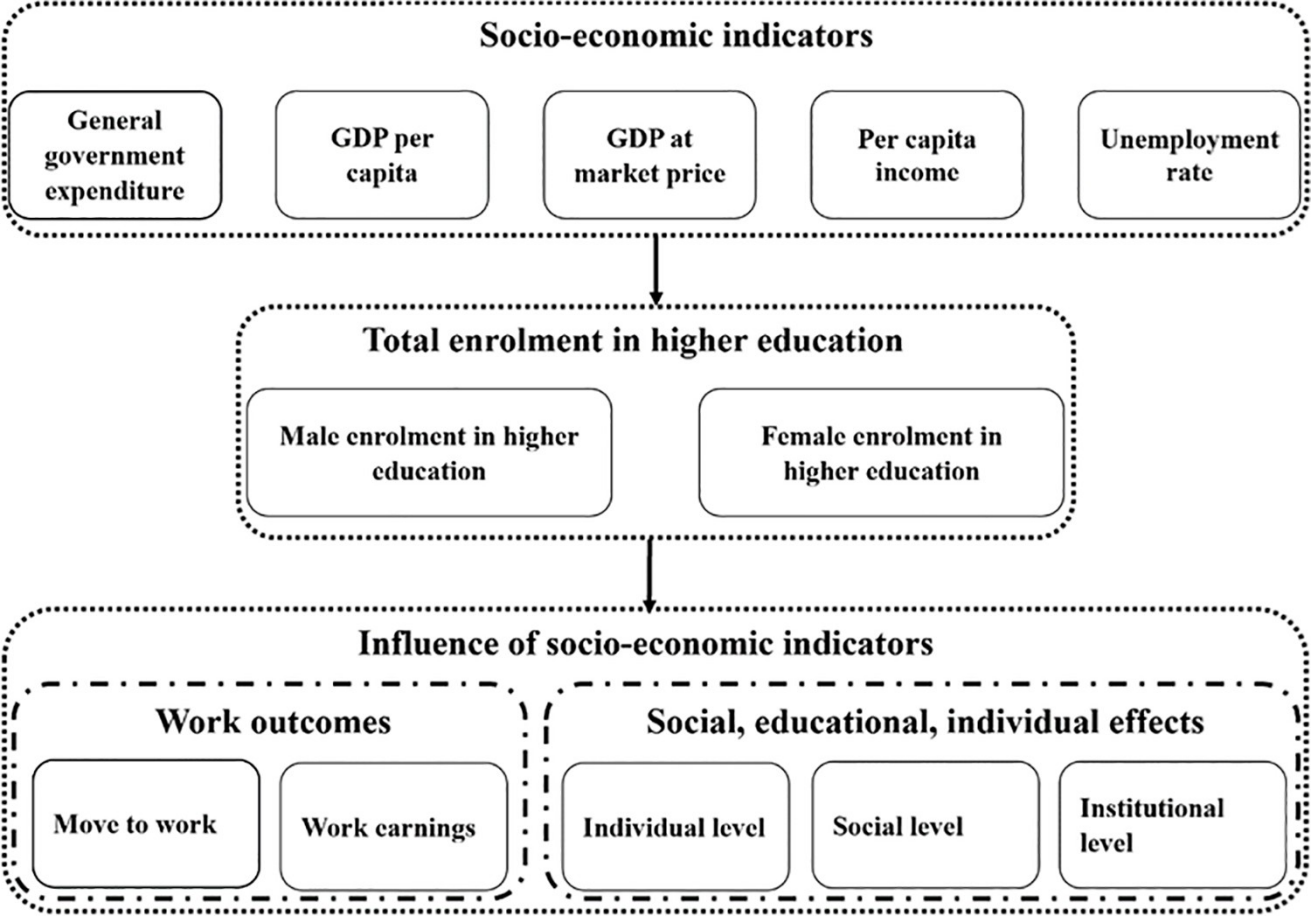
Conceptual framework of the study.

The study was intended to assess the impacts of socio-economic factors (general government expenditure, GDP per capita, GDP at market price, unemployment rate, and per capita income) on enrollment (total, male, and female) in higher education institutions of Pakistan. Socio-economic factors were selected from UNESCO and the Ministry of Finance (MOF) Government of Pakistan. The study adopted a descriptive approach to investigate the controlling effect of socio-economic factors on the students’ enrollment in higher education. The descriptive approach was selected because it enables the researchers to study the elements in their natural environment without necessarily manipulating or controlling them. The considerations and conclusions of this study will potentially be used as input for the ongoing discussion to enhance the efficiency of socio-economic factors for improving students’ enrollment in higher education institutions of Pakistan. The conceptual framework of the study is shown in Fig 1.

### 2.2 Data collection

The data used in this study were collected from secondary data sources. The secondary data regarding socio-economic factors and total enrollment, male enrollment, and female enrollment of 15 years (2001–02 to2015-16) from UNESCO, and MOF, and the Institute of Social and Policy Science (I-SAPS), to investigate the influence of socio-economic factors on enrollment in higher education in Pakistan.

The dataset provides detailed information on the influence of socio-economic factors on the total enrollment of students, as well as on male enrollment and female enrollment in all higher education institutions of Pakistan. Enrollment rates are total in numbers and measure the total number of students in the male and female sectors of higher education.

### 2.3 Analytical methods

To describe the magnitude of the relations among socio-economic indicators and students’ enrollment in higher education, Pearson’s correlation coefficients (r) were performed. Principle component analysis (PCA) based on the correlation matrix to identify influential socio-economic indicators for identifying their impact on enrollment in higher education was also performed. PCA biplots based on the two main components were plotted separately to show the relationships among studied socio-economic indicators and students’ enrollment. To group the socio-economic indicators based on all studied indicators, cluster analysis was performed using standardized data with Ward’s method. To determine the best cutoff point of the dendrogram, the canonical discriminant analysis was used. The aforementioned analysis was performed using the JMP Pro version 15. (SAS Institute Inc., Cary, NC, 1989–2019). For understanding the relationships between socio-economic indicators and students’ enrollment and recognizing indicators that play the most important role in students’ enrollment in higher education, stepwise regression analysis (IBM SPSS Version19.0 (IBM Corporation, Armonk, NY, USA) with respect to student’s enrollment (total, male, and female) as a dependent variable and socio-economic indictors as the independent variable was performed, and the variables with the highest share in justifying the variations of the dependent variable were identified.

The student’s enrollment (total, male, female) was computed as the total of the enrollment for fifteen years in Eq 1 below.


$$Y_{Index}=(Enrollment\;(t),\;Enrollment\;(m),\;and\;Enrollment\;{\left(f\right)}_{Year1}+Enrollment\;(t),\;Enrollment\;(m),\;and\;Enrollment\;{\left(f\right)}_{Year2}+\ldots \ldots \ldots \;Enrollment\;(t),\;Enrollment\;(m),\;and\;Enrollment\;{\left(f\right)}_{Year15})/15$$ (Eq 1)

Where;

**Y**_Index_ = Students’ enrollment (total, male, and female)

The 1^st^ general model (for predicting the relationship between socio-economic indicators and total students’ enrollment in higher education institutions in Pakistan was written in the following form as in Eq 2 below. To establish the relationship between socio-economic indicators and total students’ enrollment in higher education institutions in Pakistan, Eq 2 was modeled:

$$Y=\beta_{0}+\beta_{1}X_{1}+\beta_{2}X_{2}+\beta_{3}X_{3}+\beta_{4}X_{4}+\beta_{5}X_{5}+\varepsilon i$$ (Eq 2)

Where;

**Y** is total students’ enrollment and is a linear function of X_1_, X_2,_ X_3_, X_4,_ and X_5_ plus **ԑi** as computed from Eq 2 above.

**β**_**0**_ is the regression constant or intercept

**X**_**1-p**_ are independent variables of socio-economic indicators (general government expenditure, X_1_; unemployment, X_2_; per capita income, X_3_; GDP per capita, X_4_; GDP at market price, X_5_)

**β**_**1-p**_ are the regression coefficients/ change induced in **Y** by each **X**_**1-p**_

**ԑi** is a random variable, an error term that accounts for the variability in **Y** that cannot be explained by the linear effect of the **i** predictor variables.

The 2^nd^ general model (for predicting the relationship between socio-economic indicators and male enrollment in higher education institutions in Pakistan was written in the following form as in Eq 3 below. To establish the relationship between socio-economic indicators and male enrollment in higher education institutions in Pakistan, Eq 3 was modeled:

$$Y=\beta_{0}+\beta_{1}X_{1}+\beta_{2}X_{2}+\beta_{3}X_{3}+\beta_{4}X_{4}+\beta_{5}X_{5}+\varepsilon i$$ (Eq 3)

Where;

**Y** is total students’ enrollment and is a linear function of X_1_, X_2,_ X_3_, X_4,_ and X_5_ plus **ԑi** as computed from Eq 3 above.

**β**_**0**_ is the regression constant or intercept

**X**_**1-p**_ are independent variables of socio-economic indicators (general government expenditure, X_1_; unemployment, X_2_; per capita income, X_3_; GDP per capita, X_4_; GDP at market price, X_5_)

**β**_**1-p**_ are the regression coefficients/ change induced in **Y** by each **X**_**1-p**_

**ԑi** is a random variable, an error term that accounts for the variability in **Y** that cannot be explained by the linear effect of the **i** predictor variables.

The 3^rd^ general model (for predicting the relationship between socio-economic indicators and female enrollment in higher education institutions in Pakistan was written in the following form as in Eq 4 below. To establish the relationship between socio-economic indicators and female enrollment in higher education institutions in Pakistan, Eq 4 was modeled:

$$Y=\beta_{0}+\beta_{1}X_{1}+\beta_{2}X_{2}+\beta_{3}X_{3}+\beta_{4}X_{4}+\beta_{5}X_{5}+\varepsilon i$$ (Eq 4)

Where;

**Y** is female enrollment and is a linear function of X_1_, X_2,_ X_3_, X_4,_ and X_5_ plus **ԑi** as computed from Eq 4 above.

**β**_**0**_ is the regression constant or intercept

**X**_**1-p**_ are independent variables of socio-economic indicators (general government expenditure, X_1_; unemployment, X_2_; per capita income, X_3_; GDP per capita, X_4_; GDP at market price, X_5_)

**β**_**1-p**_ are the regression coefficients/ change induced in **Y** by each **X**_**1-p**_

**ԑi** is a random variable, an error term that accounts for the variability in **Y** that cannot be explained by the linear effect of the **i** predictor variables.

## 3. Results

### 3.1 Correlation between socio-economic factors and student’s enrollment

**Fig 2:**
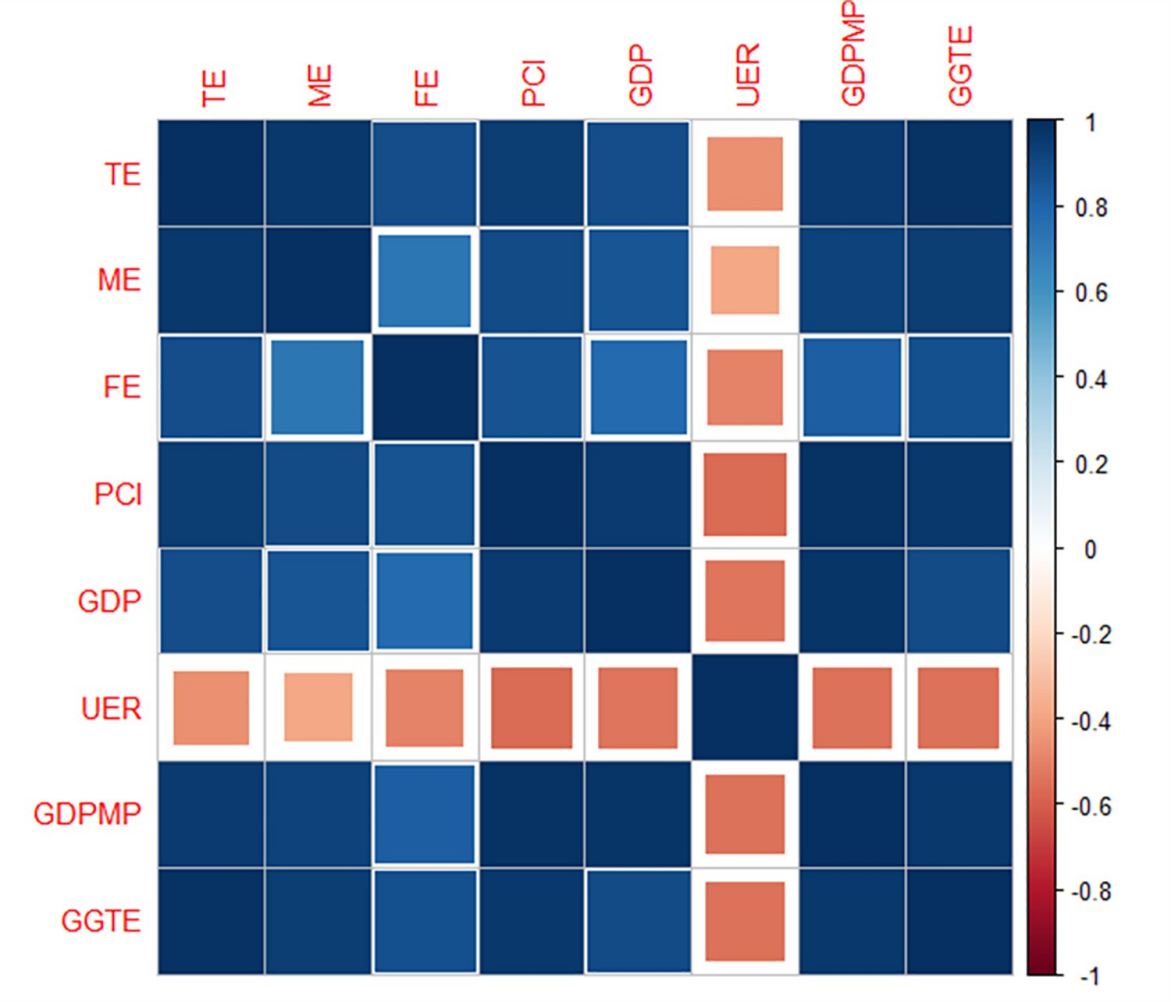
Correlation between student’s enrollment in higher education institutions of Pakistan and different socio-economic indicators using data acquired from 2001–02 to 2015–16 fiscal year. TE: Total enrollment; ME: Male enrollment; FE: Female enrollment; PCI: Parental income; GDP: Gross domestic Production; UER: Unemployment rate; GDPMP: Gross domestic production market price; and GGTE: General government expenditure.

The results of the correlation between different socio-economic indicators and students’ enrollment are depicted in Fig 2. Socio-economic indicators and students’ enrollment in higher education showed a highly significant correlation. The correlation of all the socio-economic indicators except the unemployment rate showed a positive association with students’ enrollment. The r values of the correlation between different socio-economic indicators and student’s enrollment were ranged from 0.73 to 0.99 at p< 0.01, except for the correlation between the unemployment rate and student’s enrollment, where r values were ranged from—0.39 to -0.57 at p< 0.05. The strongest correlation (r = 0.99) was observed between total enrollment and general government expenditure. In contrast, the weakest correlation (r = -0.39) was observed between male enrollment and unemployment rate.

### 3.2 Clustering analysis

**Fig 3:**
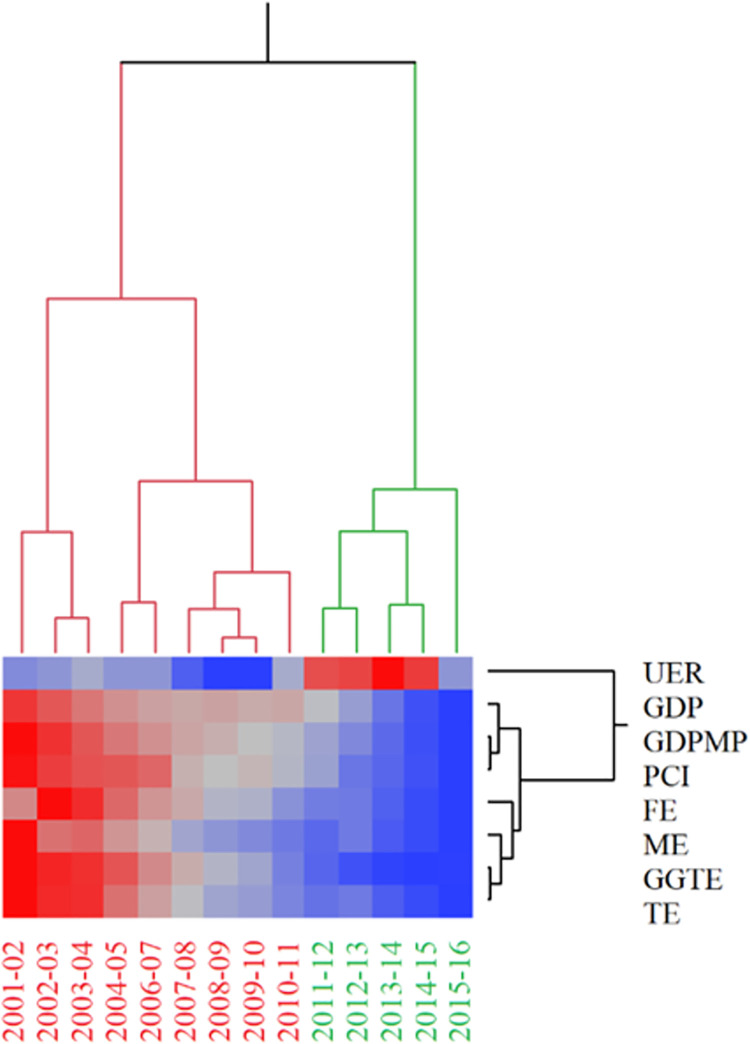
Dendrogram of cluster analysis of variables used in this study with Ward’s method.

To analyze grouping, data were subjected to hierarchical cluster analysis utilizing ward method in SAS that formed two groups. All the socio-economic indicators were positively correlated with each other except for the unemployment rate, which was negatively correlated as indicated by heatmap color codes (Fig 3). A linear decreasing trend was revealed by all factors from 2001 to 2016 whereas, the unemployment rate was decreased from 2001 to 2010 but a significant gradual increase has been observed during subsequent years showing a positive trend in the enrolment. In light of the results shown through multivariate clustering, two main clusters divide these two-time frames with distinct boundaries indicating an accelerated change in the enrollment pattern during the second era based on the data taken for 15 years. Concerning socio-economic indicators, the unemployment rate alone is forming one cluster while the rest of all factors are in the second group. The unemployment rate is affected somewhat by GDP and female enrollment in comparison to the remaining indicators.

### 3.3 Principal component analysis

**Fig 4:**
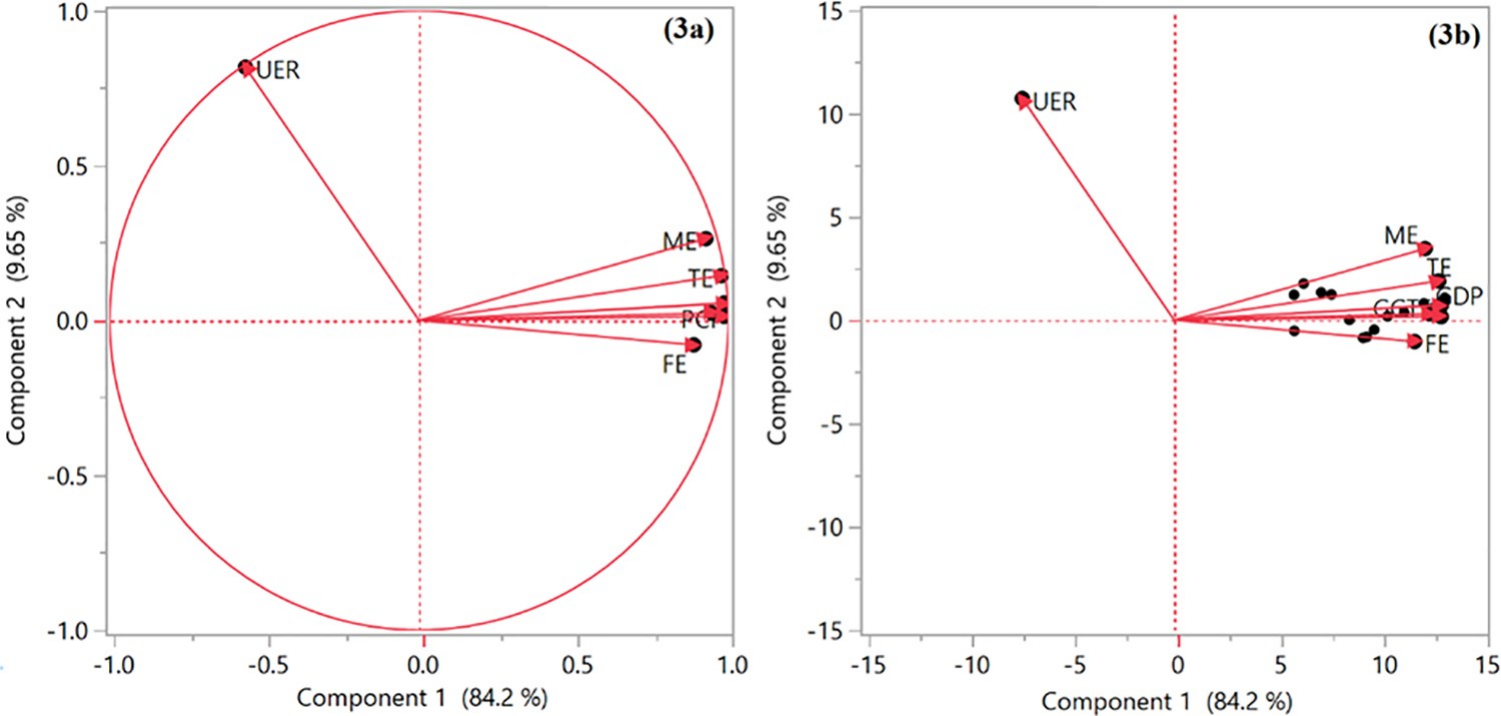
Principal component analysis and biplot showing grouping of variables used in this study. TE: Total enrollment; ME: Male enrollment; FE: Female enrollment; PCI: Parental income; GDP: Gross domestic Production; UER: Unemployment rate; GDPMP: Gross domestic production market price; and GGTE: General government expenditure.

To analyze the principal components, the matrix of correlation coefficients between variables was first calculated and then the eigenvalues and eigenvectors were extracted. The results of the principal component analysis showed that the first two components were the most influential with a cumulative contribution to the total variation of 93.85% Fig 4A. All the variables except unemployment rate had highly positive loading into the first principal component while the unemployment rate had highly positive loading into the second principal component. The relationships with respective principal components are further illustrated by the principal component biplots in Fig 4B. Accordingly, the socio-economic indicators were grouped according to the first two main components. According to the results, the plot based on the first two main components was completely consistent with the results of cluster analysis.

### 3.4 Relationship between socio-economic factors and total enrollment in higher education

The quantitative relationship between socio-economic factors (general government expenditure, unemployment, per capita income, GDP per capita (ppp constant 2011 international $), GDP at market price), and a total enrollment of students in higher education of Pakistan was established. The association between socioeconomic factors and the total enrollment of students in higher education is hypothetically feasible. The results presented in Tables 1 and 2 indicated that general government expenditure and unemployment rate have a strong and positive impact (Sig. = 0.000) on total enrollment of students in higher education institutions of Pakistan with values of correlation coefficient (R) and adjusted R^2^ ranging from 0.987 to 0.993 and 0.973 to 0.983, respectively. The positive R^2^ further implied that the model developed in this study is better than a simple baseline model.

**Table 1:** Stepwise regression for the relationships between socio-economic factors (general government expenditure, unemployment rate, per capital income, GDP per capita (PPP constant 2011 international $), and GDP at market price), and total enrollment in Pakistan during 2001–02 to 2015–16.

Model summary							
Method: Stepwise (Criteria: Probability-of-F-to-enter < = 0.05, Probability-of-F-to-remove > = 0.1).							
					ANOVA		
**Stepwise Model**	**R**	**R** ^**2**^	**Adjusted R** ^**2**^	**Std. Error of the Estimate**	**Mean Square**	**F**	**Sig.**
**1**	0.987^a^	0.975	0.973	108262.45	5843651155283	498.57	0.000^b^
**2**	0.993^b^	0.986	0.984	84270.69	2955401215387.91	416.16	0.000^c^

Source: The ministry of finance (MOF) Pakistan, Institute of Social and Policy Science (I-SAPS), Pakistan, UNESCO (2018).

a. Predictors: (Constant), General Government Expenditure.

b. Predictors: (Constant), General Government Expenditure, Unemployment Rate.

c. Dependent Variable: Total Enrollment in Higher Education.

**Table 2:** Regression coefficients for the relationship between total enrollment, socio-economic factors (general government expenditure, unemployment rate, per capital income, GDP per capita (PPP constant 2011 international $), and GDP at market price) of Pakistan during 2001–02 to 2015–16.

Model	Indicators:	Unstandardized Coefficients		Standardized Coefficients		
	Methods: (Criteria: Probability-of-F-to enter < = 0.5,					
	Probability-of-F-to-remove > = 0.1).	B	Std. Error	Beta	t	Sig.
**1**	(Constant)	346932.29	54777.77		6.33	0.000
	General Government expenditure Education	3.60	0.000	0.99	22.33	0.000
**2**	(Constant)	-284038.29	209574.43		-1.36	0.200
	General Government expenditure	3.89	0.000	1.07	24.62	0.000
	Unemployment Rate	83835.77	27263.32	0.13	3.08	0.010

Dependent Variable: Total Enrollment in Higher Education.

Two models were generated using stepwise regression, where the F probability to enter and remove of ≤ 0.05 and ≥ 0.1, respectively were used at a confidence interval of 95% (Table 1). Both models were highly significant, yet model 2 gave a more reliable prediction regarding the relationship between socio-economic and demographic factors, and the total enrollment of students in higher education, as it includes the maximum form of both factors while predicting enrollment in higher education. Lower values of the standard error of the estimate (108262 to 84271), F values (499 to 416), and higher values of adjusted R^2^ (0.97 to 0.98) for model 2 further indicated its robustness for the prediction of students’ enrollment. Model two explained 98% of the total variance associated with the relationship between socio-economic factors, and total students’ enrollment in higher education institutions of Pakistan. Our results indicated that socio-economic factors can serve as a significant predictor of total students’ enrollment in higher education institutions in Pakistan.

The coefficients of the prediction models between socio-economic factors and students’ enrollment are presented in Table 2. The specific beta coefficient values indicated that general government expenditure and unemployment rate influenced the total enrollment of students in higher education institutions in Pakistan. The results revealed that -284038 (constant) would be enrolled to attain higher education without any form of socio-economic factors. It is evident from the values of standardized coefficients, general government expenditure is the form of socio-economic factors that had a significantly positive (p≤0.01) impact on total enrollment of students with a positive beta value of 1.068. In contrast, unemployment is the form of socio-economic factors that have a less significant positive (p ≤ 0.01) impact on total student enrollment in higher education with a positive beta value of 0.133. Therefore, the predictive model for the relationship between socio-economic factors, and total student’s enrollment in higher education in Pakistan takes the form of:

Totalstudents’enrollment=1.068generalgovernmentexpenditure+0.133unemployment (Eq 5)

### 3.5 Relationship between socio-economic factors and male enrollment in higher education

The quantitative relationship between socio-economic factors (general government expenditure, unemployment rate, per capita income, GDP per capita (ppp constant 2011 international $), GDP at market price), and male enrollment of students in higher education of Pakistan was established. The association between socioeconomic factors and male enrollment of students in higher education is hypothetically feasible. The results presented in Tables 3 and 4 indicated that general government expenditure and unemployment rate have a robust and positive impact (Sig. = 0.000) on male enrollment of students in higher education institutions of Pakistan with values of correlation coefficient (R) and adjusted R^2^ ranging from 0.950 to 0.965 and 0.895 to 0.919, respectively. The positive R^2^ further implied that the model developed in this study is better than a simple baseline model. Two models were generated using stepwise regression, where the F probability to enter and remove of ≤ 0.5 and ≥ 0.1, respectively were used at a confidence interval of 95% (Table 3). Both models were highly significant, yet model 2 gave a more reliable prediction regarding the relationship between socio-economic factors, and male enrollment of students in higher education, as it includes maximum forms of factors while predicting male enrollment in higher education. Lower values of the standard error of the estimate (141960 to 124489), F values (119.8 to 80.3), and higher values of adjusted R^2^ (0.89 to 0.92) for model 2 further indicated its strength for the prediction of male students’ enrollment. Model 2 explained 92% of the total variance associated with the relationship between socio-economic factors, and male students’ enrollment in higher education institutions of Pakistan. Our results indicated that socio-economic factors can serve as a significant predictor of male students’ enrollment in higher education institutions in Pakistan.

**Table 3:** Stepwise regression for the relationships between male enrollment, socio-economic factors (general government expenditure, unemployment rate, per capital income, GDP per capita (PPP constant 2011 international $), and GDP at market price) of Pakistan during 2001–02 to 2015–16.

Model summary							
Method: Stepwise (Criteria: Probability-of-F-to-enter < = 0.05, Probability-of-F-to-remove > = 0.1).							
					ANOVA		
**Stepwise Model**	**R**	**R** ^**2**^	**Adjusted R** ^**2**^	**Std. Error of the Estimate**	**Mean Square**	**F**	**Sig.**
**1**	0.950^a^	0.902	0.895	141960.19304	2414206692070.740	119.796	0.000^b^
**2**	0.965^b^	0.931	0.919	124488.87610	1245110991048.990	80.343	0.000^c^

Source: The ministry of finance (MOF) Pakistan, Institute of Social and Policy Science (I-SAPS), Pakistan, UNESCO (2018).

a. Predictors: (Constant), General Government Expenditure.

b. Predictors: (Constant), General Government Expenditure, Unemployment.

c. Dependent Variable: Male Enrollment in Higher Education.

**Table 4:** Regression coefficients for the relationship between male enrollment, socio-economic factors (general government expenditure, unemployment rate, per capital income, GDP per capita (PPP constant 2011 international $), and GDP at market price) of Pakistan during 2001–02 to 2015–16.

Model	Indicators: Methods: (Criteria: Probability-of-F-to enter < = 0.5,	Unstandardized Coefficients		Standardized Coefficients		
	Probability-of-F-to-remove > = 0.1).	B	Std. Error	Beta	t	Sig.
**1**	(Constant)	105271.60	71827.88		1.47	0.167
	General Government Expenditure	2.31	0.000	0.95	10.95	0.000
**2**	(Constant)	-566052.84	309593.82		-1.83	0.092
	General Government Expenditure	2.63	0.000	1.08	11.25	0.000
	Unemployment	89197.51	40274.74	0.21	2.22	0.047

Dependent Variable: Male Enrollment in Higher Education.

The coefficients of the prediction models between socio-economic factors and male students’ enrollment are presented in Table 4. The specific beta coefficient values indicated that general government expenditure and unemployment rate influenced the male enrollment of students in higher education institutions in Pakistan. The results revealed that -566053 (constant) would be enrolled to attain higher education without any form of socio-economic factors. It is evident from the values of standardized coefficients, general government expenditure is the form of socio-economic factors that had a significantly positive (p≤0.01) impact on male enrollment of students with a positive beta value of 1.079. In contrast, unemployment is the form of socio-economic factors that have a less significant positive (p≤0.01) impact on male student enrollment in higher education with a positive beta value of 0.213. Therefore, the predictive model for the relationship between socio-economic factors, and male student’s enrollment in higher education in Pakistan takes the form of:

Maleenrollment=1.079generalgovernmentexpenditure+0.213unemployment (Eq 6)

### 3.6 Relationship between socio-economic factors and female enrollment in higher education

The quantitative relationship between socio-economic factors (general government expenditure, unemployment rate, per capita income, GDP per capita (ppp constant 2011 international $), GDP at market price), and female enrollment of students in higher education of Pakistan was established. The association between socioeconomic factors and female enrollment of students in higher education is hypothetically feasible. The results presented in Tables 5 and 6 revealed that only general government expenditure has a robust and positive impact (Sig. = 0.000) on enrollment of the female student in higher education institutions of Pakistan with values of correlation coefficient (R) 0.88, and adjusted R^2^ 0.75. The positive R^2^ also implied that the model developed in this study is better than a simple baseline model.

**Table 5:** Stepwise regression for the relationships between female enrollment, socio-economic factors (general government expenditure, unemployment rate, per capital income, GDP per capita (PPP constant 2011 international $), and GDP at market price) of Pakistan during 2001–02 to 2015–16.

Model summary							
Method: Stepwise (Criteria: Probability-of-F-to-enter < = 0.05, Probability-of-F-to-remove > = 0.1).							
					ANOVA		
**Stepwise Model**	**R**	**R** ^**2**^	**Adjusted R** ^**2**^	**Std. Error of the Estimate**	**Mean Square**	**F**	**Sig.**
**1**	0.88^a^	0.77	0.75	132447.491	745792425590.93	42.52	0.000^b^

Source: The ministry of finance (MOF) Pakistan, Institute of Social and Policy Science (I-SAPS), Pakistan, UNESCO (2018).

a. Predictors: (Constant), General Government Expenditure.

b. Dependent Variable: Female Enrollment in Higher Education.

**Table 6:** Regression coefficients for the relationship between female enrollment, socio-economic factors (general government expenditure, unemployment rate, per capital income, GDP per capita (PPP constant 2011 international $), and GDP at market price) of Pakistan during 2001–02 to 2015–16.

Model	Indicators: Methods: (Criteria: Probability-of-F-to enter < = 0.5,	Unstandardized Coefficients		Standardized Coefficients		
	Probability-of-F-to-remove > = 0.1).	B	Std. Error	Beta	t	Sig.
**1**	(Constant)	24166.69	67014.73		3.61	0.003
	General Government Expenditure	1.29	0.000	0.88	6.52	0.000

Dependent Variable: Female Enrollment in Higher Education.

The single model was generated using stepwise regression, where the F probability to enter and remove of ≤.05 and ≥ 0.1, respectively were used at a confidence interval of 95% (Table 5). The single model was highly significant and gave a more reliable prediction regarding the relationship between socio-economic factors, and female enrollment of students in higher education, as it includes the maximum form of both factors while predicting female enrollment in higher education. Value of the standard error of the estimate (132447), F values (43), and value of adjusted R^2^ (0.75) for a single model further indicated its strength for the prediction of female students’ enrollment. The single model explained 75% of the total variance associated with the relationship between socio-economic factors, and female students’ enrollment in higher education institutions of Pakistan. Our results indicated that socio-economic factors can serve as a significant predictor of female students’ enrollment in higher education institutions in Pakistan.

The coefficients of the prediction model between socio-economic and female students’ enrollment are presented in Table 6. The specific beta coefficient values indicated that general government expenditure influenced the female enrollment of students in higher education institutions in Pakistan. The results revealed that 241661 (constant) would be enrolled to attain higher education without any form of socio-economic factors. It is evident from the values of standardized coefficients, general government expenditure is the form of socio-economic factors that had a significantly positive (p≤0.01) impact on female enrollment of students with a positive beta value of 0.875. Therefore, the predictive model for the relationship between socio-economic factors, and female student’s enrollment in higher education in Pakistan takes the form of:

Femaleenrollment=0.875generalgovernmentexpenditure (Eq 7)

## 4. Discussion

Education being one of the most powerful media known for reducing poverty and inequality has the potential for laying the foundation for sustained economic growth, sound governance, and effective institutions [32]. Better educated nations foster civic participation, a stable democracy, and richer cultural life. Access to education for all ensures the quantity and quality of education benefit all segments of society by changing macroeconomic growth as a consequence of its influences on the labor force, governance, and the workings of most institutions. Enrollment is becoming an important social differentiator and allocator under the current scenario of development, growth, and expansion in higher education [33]. Increasing population and worsened socio-economic situations in developing countries posed negative impacts on enrollment in higher education [34]. Socio-economic background influences enrollment in higher education as students from the socially disadvantaged group are less likely to participate in higher education. Most disadvantaged students are around 6 times less likely to participate in university compared to the 20 percent most advantaged students in the UK [35]. These students are also more sensitive to the costs of education and the effect of the subsidy is larger for socially disadvantaged students [36]. Higher education expansion can be understood better as a transition from elite to mass higher education and subsequently to universal access.

Highly educated individuals as human capital is generally absorbed in national and global economies on higher paid jobs and add to the nation’s strength in the global knowledge-based economy. Theoretical and empirical evidence showed a strong and positive effect of human capital on economic growth [37]. Socio-economic indicators influence the enrollment in higher education [13] and the enrollment in higher education is generally reported to be dependent on the social and economic conditions of students and a country [20]. From an economic viewpoint, several factors are expected to influence the degree of higher education enrollment. The steady increase in socio-economic indicators including per capita income, GDP per capita, general government expenditure of a country, GDP at market price, parental income and education, and unemployment rate influence the capacity of students to receive an education that will equip them with skills to play their role as a productive citizen in different countries including those of South Asia [38, 39]. Besides, the parental income and education background also influence the educational decisions of individuals [23]. Likewise, in a high-income country, a larger number of students are attaining higher education. Income effect on higher education enrollment is positive, and students from poorer backgrounds may not be able to invest in their education [2, 12]. Country budget, subsidy for health, and opportunities for earning also increase enrollment in all levels [30]. Developed countries possibly will attract more and more students (male and female) to enroll in higher education institutions [20, 39]. These results reported in these studies are in consensus with our results, which showed that general government expenditure which depends upon the economic condition of a country significantly influences enrollment (total, male and female) in higher education institutions. Despite the equal way of educational completion by gender, women lagged behind men in occupational attainment [40]. A study conducted in Korea showed that higher levels of educational attainment had little impact on female labor force contribution [41]. In contrast in Taiwan, higher levels of education increased women’s probability of employment [42]. Additionally, future earnings, per capita income, and economic provision positively influence male enrollment [25]. The expansion of higher education was accompanied by a growing problem of graduate unemployment [43]. Previous studies showed that unemployment is a cause and a result of higher education, also support the present study which indicated that unemployment is a cause of high enrollment [5, 6, 44]. The reason is that high enrollment in higher education helps to increase the probability of unemployment rate and the unemployment rate can be easily decreased by pulling the idle workforce back into the university system. All studies discussed above are in consensus with our study except one study stating that unemployment has no significant effect on enrollment. In light of the results shown through multivariate clustering, two main clusters divide these two-time frames with distinct boundaries indicating an accelerated change in the enrollment pattern during the second era based on the data taken for 15 years. This strong correlation between enrollment and socio-economic indicators was attributed to a better socio-economic condition in Pakistan after the negative impacts of decade long war against terrorism on the socio-economic and socio-political status of the Pakistani nations. The higher spending by the government on education and higher income due to increased economic activities has resulted in higher enrollment in higher education institutions in Pakistan were in accordance with the human capital theory. Foregoing researches described numerous other indicators such as literacy rate, population, student’s test score, student-teacher ratio, and institutions have a significant impact on student’s enrollment in higher education [30]. Nevertheless, there is consensus that government financing can make available the required short-term liquidity by philanthropic credits to the students from poorer backgrounds to enhance enrollment in higher education.

## 5. Conclusion

This study attempted to appraise the influence of the socio-economic indicators (general government expenditure, unemployment, per capita income, GDP per capita (ppp), GDP at market price) on indicators on increasing participation and study decisions in higher education in Pakistan. As a result of this research, socio-economic indicators influencing students’ enrollment in higher education institutions of Pakistan were identified and their impacts on enrollment in higher education were evaluated. The results of the PCA showed that the first two components were the most influential with a cumulative contribution to the total variation of 93.85%. The results also revealed that enrollment has significant relationships with general government expenditure and unemployment rate (total and male enrollment) and general government expenditure (female enrollment). A potential restraint of the study is that the conclusions might only be justified in Pakistan and to the countries having a similar/comparable governance and higher education system. The focus of the research was only on five socio-economic indicators (general government expenditure, per capita income, unemployment rate, GDP per capita, GDP at market price) according to UNESCO, and MOF government of Pakistan without considering all socio-economic indicators from other national and international sources to explore the role of socio-economic indicators on student enrollment (total, male, and female). The models only used the socio-economic indicators and excluded all the socio demographic, educational, and governmental factors such as population, government funding, enrollments in elementary, secondary, and higher secondary education, parental income, and literacy rate that might also influence the enrollment in higher education institutions. Therefore, to amplify this broad-brush picture and to provide an in-depth understanding of the factors affecting student enrollment in higher education institutions, a more detailed analysis taking other potential factors is required. The findings will assist educationists, social scientists, and policymakers to better understand the association between social economic indicators and student enrollment in higher education for formulating future education policies for enhancing enrollment in higher education.
